# Modelling the impact of declining insecticide resistance with mosquito age on malaria transmission

**DOI:** 10.5281/zenodo.10876461

**Published:** 2015-11-14

**Authors:** Adam Saddler, Jacob C Koella

**Affiliations:** 1 Division of Biology, Faculty of Life Sciences, Imperial College London, Silwood Park Campus, Ascot, Berkshire SL5 2PZ, United Kingdom.; 2 Faculté des Sciences, Institut de Biologie, Université de Neuchâtel, Rue Emile-Argand 11, CH-2000 Neuchâtel, Switzerland.; 3 Department of Epidemiology and Public Health, Swiss Tropical and Public Health Institute, Socinstrasse, 57, CH-4002 Basel, Switzerland.; 4 Ifakara Health Institute, Box 74, Bagamoyo, Tanzania.; 5 University of Basel, Petersplatz 1, Basel 4003, Switzerland.

## Abstract

**Background:**

The evolution of insecticide resistance can lead to an increase in the entomological indicators of malaria transmission, such as mosquito survival and blood feeding rates, thus threatening efforts to control malaria. Yet, there is little evidence from the field that malaria control programmes are failing due to insecticide resistance. One explanation for this apparent contradiction is the growing evidence that insecticide resistance declines with mosquito age. Once a mosquito is first infected by *Plasmodium* parasites, it will not be able to transmit those parasites until they have undergone development, which lasts around 10 days. Thus, although the evolution of resistance in a population will enhance the survival of young mosquitoes, the insecticide may still kill old, and thus potentially infectious, mosquitoes, and thereby maintaining its efficacy in controlling malaria.

**Materials and Methods:**

The current evidence for age-related insecticide resistance is reviewed. A mathematical model is then described that predicts how the decline of resistance with the age of a mosquito will affect the intensity of transmission of malaria. The model combines the behavioural response of the mosquitoes to insecticides with an epidemiological model of malaria.

**Results:**

It was found that phenotypic resistance decreases between 1.37% to 9.71% per day, independent of the mosquito species or strain. The models suggest that a decline in resistance within this range strongly diminishes the predicted impact of insecticide resistance on the effectiveness of malaria transmission-controlling interventions.

**Conclusions:**

Our model can be used to assess the threat of insecticide-resistance for the control of malaria. The model confirms observations from the field suggesting that, even where genetically insecticide-resistant mosquitoes dominate populations, insecticides can substantially reduce the transmission of malaria.

## 1 Introduction

The evolution of insecticide resistance is threatening to undermine our efforts to control malaria through control of the mosquito vectors [1,2]. By definition, resistance allows mosquitoes to survive or avoid exposure to insecticides, leading to increased biting rates on humans and an increase in mosquito survival [3,4]. When using such entomological outcomes to predict epidemiological endpoints, a clear negative correlation between the effectiveness of insecticide-treated bed nets (ITNs) and level of insecticide resistance is seen [5]. Yet, despite the emergence of resistance soon after insecticides were first used for vector control in the 1950s, there are few examples from the field that insecticide resistance is linked to failing malaria control programmes. Resistance to pyrethroids in South Africa might have led to a rebound in malaria prevalence [6] and might have reduced the impact of indoor residual spraying (IRS) on malaria prevalence in Malawi; however, larger and more powerful studies are needed to confirm these observations [7]. By contrast, studies have shown that, despite increasing insecticide resistance [8] and even in areas with extremely high (80%) resistance [9], IRS and ITNs [10] continue to decrease the prevalence of malaria. Thus, although the evolution of resistance impedes our control of mosquitoes, it does not imply a failure to control malaria.

One factor that could help to explain this apparent contradiction is that genetically resistant mosquitoes might in some circumstances be killed by the insecticide. Environmental factors, such as temperature [11,12], food quality [13,14] and parasitism [15-17], can influence the expression of resistance in mosquitoes. Such environmental stresses therefore decrease the number of mosquitoes that survive the exposure to an insecticide below what is expected from the proportion of mosquitoes that are genetically resistant. Therefore, control measures might be able to impact malaria transmission even in situations in which a high proportion of the mosquito population is genetically resistant.

One of the most influential factors affecting the phenotypic expression of resistance might be that resistance declines with the age of mosquitoes. Such age-specific resistance has been documented in several mosquito species and for several resistance mechanisms, in laboratory experiments and under field conditions [14,18-25] (Table 1). The decline appears to be in part due to a decline in expression of the detoxification genes and enzymes associated with resistance [24]. However, this relationship is not always observed [19,21]; indeed, resistance can decline with age even when the resistance mechanism is a target site mutation, which involves a permanent change to the mosquito nervous system (Table 1) and therefore does not involve detoxification genes. In such cases, resistance might decline with age if senescence reduces the general condition or energy reserves of mosquitoes. Some support for this notion is that blood feeding can negate the decline in resistance with age [21], but this has not been repeated in other studies [19].

**Table 1.** Studies that measured insecticide resistance with mosquito age.

Study	Mosquito species and strain	Mechanism	Insecticide	Daily reduction in resistance*	Letter in Figure 1
Chouaibou *et al.*, 2012	*An. gambiae* (field-collected)	Unknown	Deltamethrin Permethrin DDT Propoxur	9.55 9.71 0.02 3.51	A B C D
Christian *et al.*, 2011	*An. funestus* (FUMOZ-R)	Monooxygenase (P450) detoxification	Permethrin	3.29	E
Curtis and Hodjati, 1999	*An. stephensi* (DUB234) *An. gambiae* (RSP)	Unknown Elevated esterase and oxidase	Permethrin Permethrin	2.49 1.37	F G
Hunt *et al.*, 2005	*An. funestus* (FUMOZ-R)	Monooxygenase (P450) detoxification	Lambda-cyhalothrin	4.09	H
Jones *et al.*, 2012	*An. gambiae* (S-form, field-collected)	Kdr	Deltamethrin (two tests)	4.92 3.00	- -
Kulma *et al.*, 2013	*An. gambiae* (ZAN/U)	Elevated GSTe2	DDT	8.30	I
Lines and Nassor, 1991	*An. gambiae* (field-collected)	Unknown	DDT	7.08	J
Rajatileka *et al.*, 2011	*An. gambiae* (ZAN/U) (AKRON) (RSP)	Elevated GSTe2 Kdr, ace1-R mutation Kdr and elevated GSTe2	DDT Bendiocarb DDT Bendiocarb DDT	2.27 2.09 1.91 2.64 0.55 (increase)	K L M N O
Xu *et al.*, 2014	*An. sinesis* (field-collected)	Kdr, elevated P450 monooxygenase, elevated GST	Deltamethrin	1.41	-

**Figure 1.**
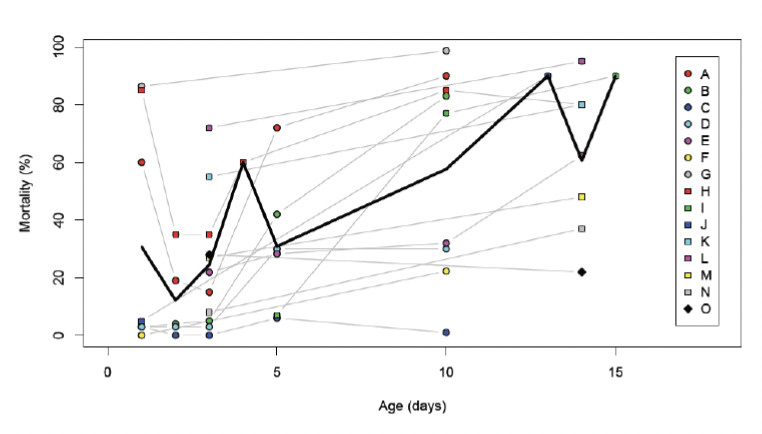
Data of studies showing the effect of age on the mortality of adult anopheline mosquitoes caused by exposure to insecticides. Coloured symbols and thin lines represent the data from individual studies. Letters correspond with those shown in Table 1. The thick line represents the average mortality of the experiments. In studies in which exact data points were unavailable, estimates were taken from figures.

* Daily reduction in resistance estimates were calculated from the linear regression slopes for each of the studies. In two studies, A and H, resistance increased dramatically in the first two days after emergence; therefore, day 1 data, for these studies, was ignored. The studies for which there were only data for mosquitoes older than 15 days were not included in Figure 1.

Whatever the mechanism, a decline of resistance with age has important epidemiological consequences. Before a malaria parasite can be transmitted, it needs to develop inside the mosquito, a process that lasts at least 10 days. Therefore, by the time genetically resistant mosquitoes are old enough to transmit malaria, they might have reached an age at which they may be killed by the insecticide. Thus, although the evolution of resistance in a population will enhance the survival of young mosquitoes, the insecticide might still kill old, and potentially infectious, mosquitoes and thus maintain to some degree its efficacy in controlling malaria.

Here, we first review the available evidence on age-specific insecticide resistance of anopheline mosquitoes (Table 1). We then describe a mathematical model that combines the behavioural response of the mosquitoes to insecticides with an epidemiological model of malaria to predict how the decline of resistance with mosquito age will affect the intensity of malaria transmission. The behavioural sub-model, following [26], is used to calculate the mortality caused by insecticides at each gonotrophic cycle after adult mosquito emergence. This value is then introduced into the epidemiological sub-model, adapted from [27], to calculate an entomological indicator of malaria transmission: the number of infectious bites expected from an emerged adult mosquito.

## 2 Materials and methods

### 2.1 Review of age-specific resistance

To find relevant data, we searched PubMed with the keywords ‘age’, ‘insecticide’, ‘resistance’ and ‘mosquito’ and then eliminated the studies that did not give any useful data (e.g. those that estimated mortality at only one age). Among the remaining studies, several investigated the time to knockdown as a measure of resistance [20,28,29]. Although these showed a decline of resistance with age, we excluded the knockdown-data from our analysis. Finally, the initially selected publications were searched for relevant references. This resulted in the data of 18 experiments in nine studies (see Table 1) that measured insecticide-induced mortality of anopheline mosquitoes as they age. As an indicator of the age-related decline of resistance, we used the slope of the regression of the average age-specific mortality over age. Because of the limited data available, we calculated the slope with the pooled data, thus combining different levels of resistance, different species and strains, as well as different insecticides. Our indicator is therefore not a quantitative measure of age -specific resistance, but a crude estimate of how resistance decreases with age.

### 2.2 Mathematical model

We calculate our measure of the intensity of transmission – the average number of infectious bites by a mosquito – by combining a description of the mosquito’s feeding cycle [26] and a description of the infection dynamics in mosquitoes [27].

*Feeding cycle*. First, mosquitoes search for hosts until they are successful or die. At each biting attempt, they search for a human, with probability *Q*, and an animal with probability 1-*Q*. If they search for a human, they encounter an insecticide-treated house with probability *j.* Mosquitoes are then either repelled and repeat the host-searching cycle at a probability *r*, or they are not repelled and successfully feed with probability (1-*r*)*s*, or they are not repelled and then killed by the insecticide with probability (1-*r*)(1-*s*). With these assumptions, the probability of achieving a successful bite at a single feeding attempt is:

$$1-Q\varphi \left[1-\left(1-r\right)s\right]$$

If the mosquito does not obtain a blood meal, it will start a new feeding attempt, and repeat this until it is successful or dies. The mosquito is therefore successful, if it succeeds on its first attempt, or it is repelled once and succeeds on its second attempt, or it is repelled twice and then succeeds on its third attempt, etc. We assume that each time the mosquito is repelled and attempts to feed again, it will encounter an additional risk of death *μr*. Thus, we then calculate the probability of feeding success as a geometric series [30]:

$$\left[1-Q\varphi \left[1-\left(1-r\right)s\right]\right]{\sum_{n=0}^{\infty }\left(Q\varphi r\left(1-\mu_{r}\right)\right)}^{n}$$

Second, once mosquitoes have successfully fed, the mosquitoes survive the remainder of the gonotrophic cycle with probability (1 – μ)^r^, where μ is the daily background mortality and τ is the length of the gonotrophic cycle of mosquitoes that are not repelled by an insecticide [27]. The probability of surviving a gonotrophic cycle (the combination of feeding-related and feeding-independent mortality) is thus:

$$\begin{array}{c} \rho ={\left(1-\mu \right)}^{\tau }\left[1-Q\varphi \left[1-\left(1-r\right)s\right]\right]{\sum_{n=0}^{\infty }\left(Q\varphi r\left(1-\mu_{r}\right)\right)}^{n}\\ ={\left(1-\mu \right)}^{\tau }\frac{\left[1-Q\varphi \left[1-\left(1-r\right)s\right]\right]}{1-Q\varphi r\left(1-\mu_{r}\right)} \end{array}$$

Note that this equation differs from equations by [26] in two ways: first, the parameters are defined differently so that the form of the equation is different, allowing easier integration of the survival function and infection dynamics. Second, we assume that the gonotrophic cycle is not prolonged by repeated host searches. A comparison of our equation with those in [26] showed that this is a good approximation except for extreme situations, e.g. if each search for a host lasts a long time or if coverage is close to 100% and mosquitoes are strongly anthropophilic (so that very many searches are necessary).

As we are interested in the effect of age on the sensitivity to insecticides, we further assume that survival is a function of age and thus replace *s* by *s_i_* and *p* by *p_i_*, where *i* is age in terms of gonotrophic cycles. To simplify the analyses (and as suggested by the data in Table 1), we assume that sensitivity increases linearly with age (analyses that are not shown suggest that other age-specificities, e.g. that resistance decreases exponentially with age, give similar conclusions).

*Infection dynamics.* Assuming, for simplicity, that all mortality associated with the gonotrophic cycle takes place before the bite takes place, if the survival during a mosquito’s *i*-th gonotrophic cycle is *pi*, the probability that a mosquito becomes infected at bite *z* (but not before) and survives the associated gonotrophic cycle is ${\left(1-\pi \right)}^{z-1}\pi {∐}_{i=1}^{z}p_{i}$, where π is the probability per bite that a mosquito is infected. It will then survive the developmental period of the parasite with probability ${∐}_{i=z+1}^{z+N}p_{i}$, where *N* is the duration in gonotrophic cycles of the parasite’s development. It will then survive to deliver $\sum_{x=z+N}^{\infty }l_{x}/l_{z+N}$ infectious bites, where $l_{x}=\prod_{i=0}^{x}p_{i}$ is the probability of survival from emergence to bite *x*. Thus, if we assume that each mosquito bites once during a gonotrophic cycle, the expected number of infectious bites of a mosquito, *b*, can be calculated from the product of the probability that a mosquito is infected at bite *z*, the probability that it survives the developmental period and its longevity once infectious, summed over the probability of bites causing primary infection in the mosquito. This reduces to:

$$b=\pi \sum_{z=1}^{\infty }\left\{{\left(1-\pi \right)}^{z-1}\sum_{x=z+N}^{\infty }l_{x}\right\}$$

*Parameters*. As we are not interested in a full exploration of the parameter space, but rather in an indication of possible patterns, we restrict ourselves to parameters that are typical for malaria in sub-Saharan Africa. Thus, we set μ, the daily background mortality of mosquitoes, to 0.1 [27], and we set t, the length of the gonotrophic cycle in days, to 3, independent of the exposure to the insecticide. As we assume that host searching has little cost, we assume that *μ_r_* is low and set it to 0.03. We assume that repellency, *r*, is 0.6 [26] and that biting preference for humans, *Q*, is 0.9, based on an anthropophilic species such as *Anopheles gambiae* [31]. We set the probability of infection during a given night, *π*, to 0.3 to reflect an area with intense transmission [32].

## 3 Results

### 3.1 Review of age-specific resistance

Figure 1 shows the data of the studies listed in Table 1. Of the 18 experiments, 16 showed a notable decline in resistance as mosquito age increased. In one experiment (C), mosquitoes were extremely resistant throughout their lives, with less than 5% mortality after exposure to DDT. Only one experiment indicated a small increase in resistance with mosquito age [24]. If the data in the youngest mosquitoes of the experiments in which insecticide-induced mortality dropped between 1-day old and 2-day old mosquitoes are ignored, the age-related decline of resistance ranges from 1.37% to 9.71% per day, independent of the mosquito’s species or strain, the overall level of resistance or the insecticide used. Average mortality increased from less than 20% in the youngest mosquitoes to about 90% in 2-week old mosquitoes, which reflects a 4.88% increase in mortality per day.

### 3.2 Mathematical model

**Figure 2.**
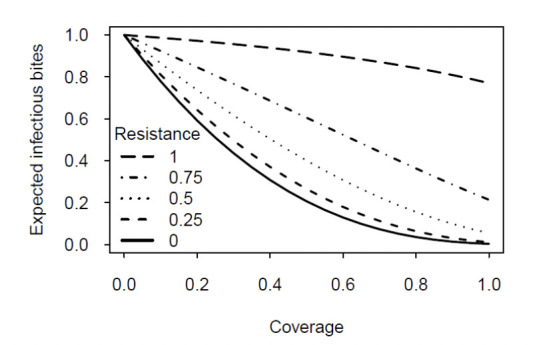
Prediction of the model for resistance that is constant over age. Each curve shows as a function of coverage the expected number of infectious bites achieved by an emerging mosquito (relative to the number in the absence of the insecticide). The level of resistance ranges from zero (all mosquitoes are killed by the insecticide: bottom curve) to one (no mosquitoes are killed: top curve).

We established a baseline by assuming that resistance is constant over age (Figure 2). Naturally, increasing resistance decreases the effect of the insecticide (i.e. increases the number of infectious bites achieved by a mosquito). Yet, insecticides decrease transmission even if mosquitoes are completely resistant (top curve in Figure 2), as the repellent action of the insecticide pushes mosquitoes to bite non-humans more frequently, reducing the likelihood that mosquitoes are infected and that they transmit the parasite.

**Figure 3.**
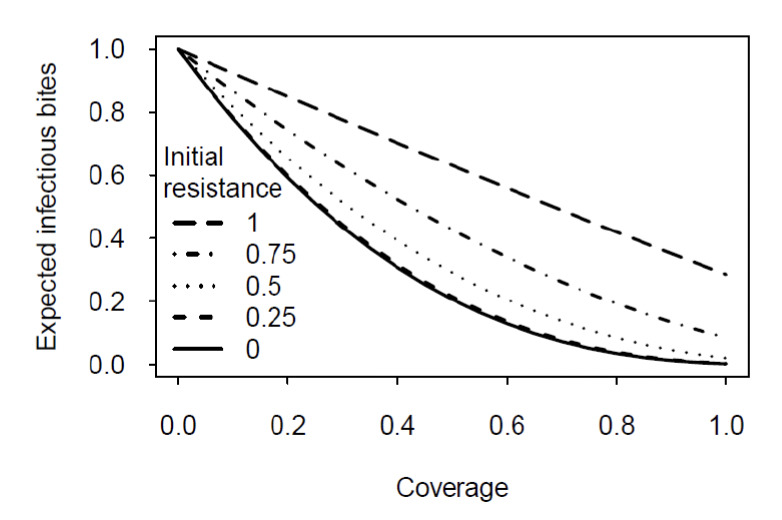
Prediction of the model for resistance that declines with age. All parameters are as in Figure 2, except that resistance declines at a rate of 5% per day.

The impact of resistance is strongly diminished, if the level of resistance decreases with age at the rate of 5% per day (the average rate suggested by the data reviewed in Figure 1). Indeed, if resistance is weak at emergence, the disappearance of resistance in old mosquitoes reduces the impact of resistance to a negligible increase of the number of infectious bites over what is found for susceptible mosquitoes (Figure 3). Even if initial resistance is complete (i.e. no newly emerged mosquitoes are killed by the insecticide), the decline over age reduces transmission to a value substantially below the level experienced with no control (zero coverage).

**Figure 4.**
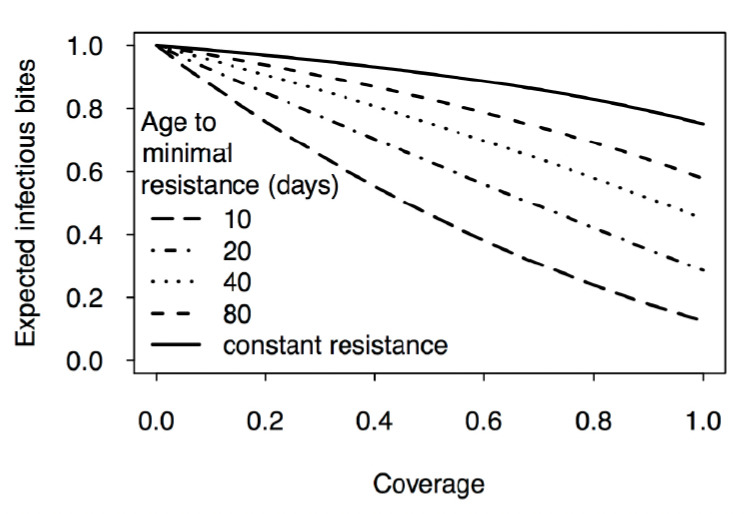
Prediction of the model for resistance that declines with age. For all curves, we assumed that the level of resistance at emergence is 100% and that the level of resistance decreases linearly with age. The decline of resistance with age ranges from zero (resistance is constant: top curve) to 10% per day (resistance disappears after 10 days: bottom curve).

Of course, the effect depends strongly on the rate of decline of resistance. For example, if mosquitoes change from fully resistant at emergence to fully susceptible at the age of 10 days, transmission is reduced by about 75%. Yet, even if resistance decreases only slowly with age, the fact that the oldest mosquitoes have little resistance has a moderate impact on the intensity of transmission (Figure 4). Thus, if resistance decreases linearly until it disappears at the age of 80 days (which is substantially higher than the average life-expectancy of 10 days assumed in our model) transmission is reduced by about 20% (Figure 4).

## 4 Discussion

The models confirm observations from the field [8-10], suggesting that, even where genetically insecticide-resistant mosquitoes dominate populations, insecticides can substantially reduce the transmission of malaria. In designing the models, our key points were (i) that genetic resistance does not necessarily reflect phenotypic resistance (i.e. the probability that an insecticide kills exposed mosquitoes) and (ii) that phenotypic resistance generally declines with the age of a mosquito. Our model suggests that resistance decreasing strongly with the age of a mosquito (Table 1) can have a significant impact on the number of infectious bites delivered by mosquitoes and thus on the intensity of transmission. Even if the time it takes for sensitivity to be restored is two to three times the average lifespan of mosquitoes (as suggested in Table 1), transmission can be reduced by at least 50% if coverage is greater than 80% (Figures 3 and 4).

As genetic resistance does not necessarily reflect phenotypic resistance any attempt to estimate the prevalence of genetic resistance in a mosquito population, whether calculated accurately by molecular tests or crudely estimated from WHO and CDC susceptibility tests [33,34], is unlikely to give reliable information about the success of genetically resistant mosquitoes in the field. Success of these mosquitoes will not only be determined by the intensity of the resistance mechanisms they carry [35] but also by mosquito age, mosquito condition and local environmental conditions. A recent meta-analysis of studies on insecticide-resistant mosquitoes showed that, although there was a moderate effect of resistance on mortality, ITNs consistently cause higher mortality and better prevent blood feeding than untreated nets [36]. This corroborates the field trials suggesting that ITNs can still reduce malaria transmission in populations of resistant mosquitoes [8-10].

The impact of resistance on malaria transmission is, however, an inherently difficult relationship to measure, as it would require very good entomological and resistance monitoring and thorough disease surveillance; yet, a new multi-country study aims to do just that [37]. As stated, basic entomological indicators such as mosquito numbers and blood feeding rates might give a false impression of changes in malaria transmission, as the numbers of young mosquitoes might increase, whereas older malaria-transmitting mosquitoes remain controlled. A better entomological indicator for field studies might therefore be the sporozoite rate (the percentage of mosquitoes with sporozoites in their salivary glands). Estimates of the age structure and longevity of resistant mosquitoes in the field, using techniques such as mark-recapture, will also provide useful information on the longevity, and thus ability, of the local mosquito populations to transmit malaria.

The decline of resistance with age might not be the sole mechanism maintaining the efficacy of ITNs in the face of widespread resistance. Additional stressors, such as mosquito nutrition [11,12] or parasitism [13-15], which often has a greater impact on old and potentially malaria-transmitting mosquitoes [38,39], can also influence mosquito susceptibility to insecticides. Furthermore, costs of carrying genes for resistance can lead to a reduction in mosquito survival [40,41], thus potentially affecting the ability of the mosquitoes to transmit malaria. It has been shown that a strain of *An. gambiae* with *kdr* resistance has lower malaria parasite loads when compared to susceptible mosquitoes; however, the same study indicated that the resistant mosquitoes had higher initial infection rates (percentage of mosquitoes that are infected after a blood meal), leading to higher sporozoite prevalence [42]. A higher sporozoite prevalence raises concern that resistant mosquitoes are potentially more effective vectors than sensitive mosquitoes; however, differences in infection rates of wild-caught mosquitoes were not observed [43].

The impact of insecticide resistance on malaria transmission is therefore very complex and is likely to vary widely due to many factors, including the specific mechanism of resistance, the mosquito species, local environmental conditions and all the different permutations. This is epitomised by the high heterogeneity seen in the efficacy of using ITNs in resistant mosquito populations [36]. A good review on the potential relationship between vector capacity and insecticide resistance is given here [44], and it highlights how little we know and the vast amount of research needed in this area.

Thus, although insecticide resistance is likely to increase the number of young mosquitoes, which might precipitate increased biting rates in the field [3,4], there are a number of reasons as to why this might not materialise in an increase in malaria transmission. In particular, the ability of the insecticide to kill the mosquitoes old enough to transmit malaria means that effective malaria control can be maintained even in areas with high levels of resistance.

## 5 Conclusions

In summary, our model can be used to assess the threat of insecticide resistance for the control of malaria. It suggests that the common observation that insecticide-based interventions continue to control malaria despite widespread resistance might be due to a decline of the phenotypic expression of resistance with the age of a mosquito. Indeed, any reduction in the phenotypic expression in resistance, whether it is due to the environment or changes with age, will reduce the probability that a resistant mosquito survives to become infectious. Whereas WHO susceptibility tests can identify the presence of resistance in a population, studies identifying the mortality rate of resistant mosquitoes in the field are needed, together with detailed mathematical modelling to assess the real threat resistance poses for malaria control.
